# Geologically calibrated mammalian tree and its correlation with global events, including the emergence of humans

**DOI:** 10.1002/ece3.10827

**Published:** 2023-12-19

**Authors:** Soichi Osozawa

**Affiliations:** ^1^ Faculty of Science, Institute of Geology and Paleontology Tohoku University Sendai Japan

**Keywords:** base substitution rate, BEAST v1.10.4, environmental changes, fossil calibration, mammals

## Abstract

A robust timetree for Mammalia was constructed using the time calibration function of BEAST v1.10.4 and MEGA 11. The analysis involved the application of times of the most recent common ancestors, including a total of 19 mammalian fossil calibration ages following Benton et al. (*Palaeontologia Electronica*, 2015, 1–106) for their minimum ages. Additionally, fossil calibration ages for *Gorilla*, *Pan*, and a geologic event calibration age for otters were incorporated. Using these calibration ages, I constructed a geologically calibrated tree that estimates the age of the *Homo* and *Pan* splitting to be 5.69 Ma. The tree carries several significant implications. First, after the initial rifting at 120 Ma, the Atlantic Ocean expanded by over 500 km around Chron 34 (84 Ma), and vicariant speciation between Afrotheria (Africa) and Xenarthra (South America) appears to have commenced around 70 Ma. Additionally, ordinal level differentiations began immediately following the K–Pg boundary (66.0 Ma), supporting previous hypothesis that mammalian radiation rapidly filled ecological niches left vacant by non‐avian dinosaurs. I constructed a diagram depicting the relationship between base substitution rate and age using an additional function in BEAST v1.10.4. The diagram reveals an exponential increase in the base substitution rate approaching recent times. This increased base substitution rate during the Neogene period may have contributed to the expansion of biodiversity, including the extensive adaptive radiation that led to the evolution of *Homo sapiens*. One significant driving factor behind this radiation could be attributed to the emergence and proliferation of C4 grasses since 20 Ma. These grasses have played a role in increasing carbon fixation, reducing atmospheric CO_2_ concentration, inducing global cooling, and initiating Quaternary glacial–interglacial cycles, thereby causing significant climatic changes.

## Graphical abstract

A robust minimum age tree shows post K–Pg Placentalia radiation, and subsequent extensive radiation connected to exponentially increased base substitution (mutation) rate including generation of Homo sapiens. This Neogene radiation may have been triggered by expansion of C4 grasses that decreased atmospheric CO_2_, resulting in global cooling and initiation of Quaternary glacial–interglacial cycles.

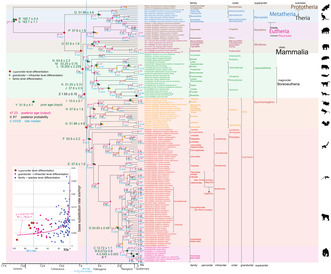

## 1 INTRODUCTION

The current DNA sequences result from base substitutions, and historical sequences can be reconstructed using MEGA 11 software (employing maximum parsimony; Tamura et al., 2021). Alternatively, a phylogenetic tree constructed from the current DNA sequences offers a visual representation of evolutionary history, where the splits in the tree indicate the divergence and independent evolution of distinct populations (Upham et al., 2021).

It is important to highlight that such a tree represents relative timescales. Establishing an absolute timescale requires calibration using reliable geological data for specific nodes. Rate dating, utilizing a molecular clock model, can estimate the age of the tree by assuming a relatively constant base substitution rate (Ronquist et al., 2019). Adopting this assumption of a strict clock model facilitates the acquisition of a dated phylogeny (Brower, 1994; van Tuinen & Hedges, 2016). The current dating approach employs node dating and considers the relaxed clock model, rather than total‐evidence tip dating (Ronquist et al., 2019).

**FIGURE 1:**
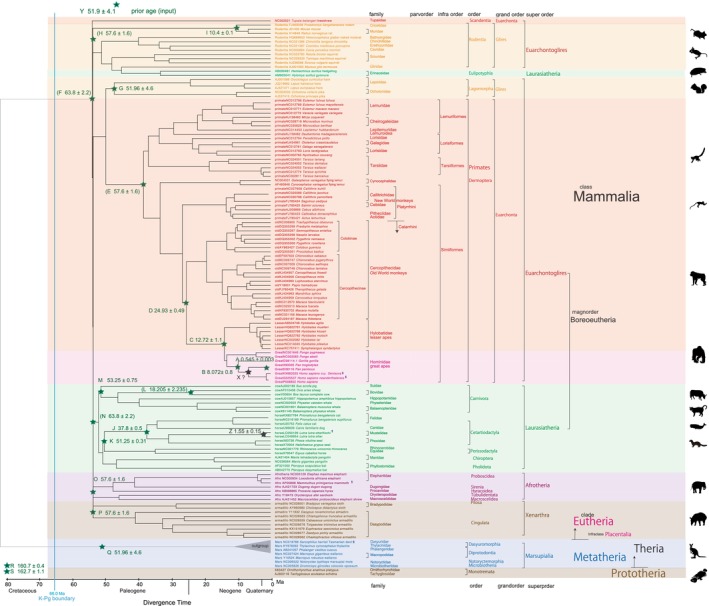
Mammalian PI timetree built by MEGA 11. Fossil calibrations from A to S are solely by minimum ages. Parenthesized E, F, H, and N are not applied, because, for example, Erinaceidae is excluded from the Laurasiatheria clade with a crown node age of N 63.8 ± 2.2 Ma.

**FIGURE 2:**
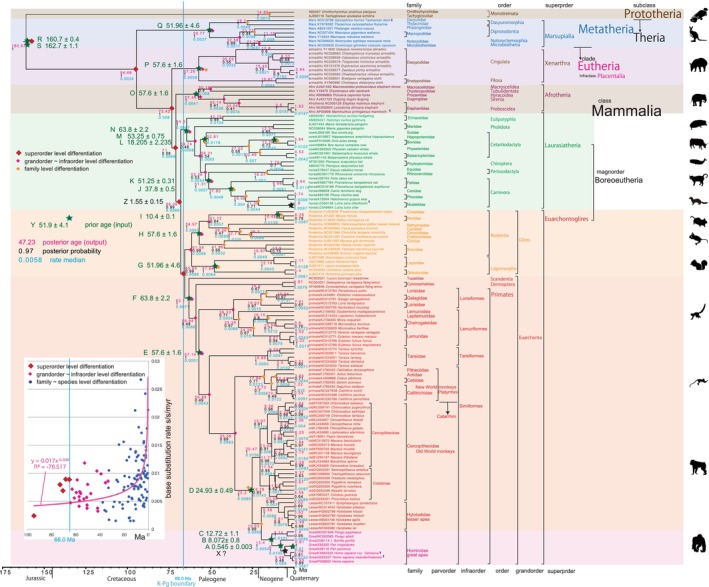
Mammalian BI timetree built by BEAST v1.10.4. Fossil calibrations from A to S are solely by minimum ages. *Pan*‐*Homo* splitting date was not calibrated, but expressed as X, and estimated at 5.69 Ma. Geological event calibration: point Z. Illustrations were downloaded from silhouette AC. Inset: Base substitution rate (= rate median shown at each node; mutations per bp per million years) versus age (= posterior age shown at each node) diagram. Heavy red curve with equation: trendline drawn by Excel function.

**FIGURE 3:**
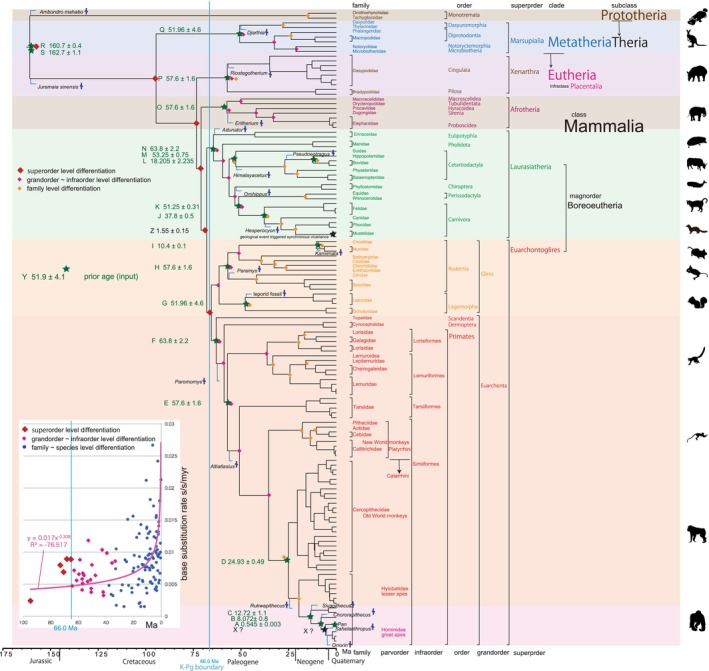
Mammalian timetree built by BEAST v1.10.4, simplified from Figure 2. Fossil ages with cross marks to adjust minimum ages (Benton et al., 2015) are shown close to each calibration point.

As an initial goal, I took the opportunity to reassess the relationships within Mammalia by conducting comprehensive molecular phylogenetic analyses. A total of 133 species were considered, encompassing 66 species from the Primate order, in addition to representatives from 56 families and 23 orders. The analysis utilized whole mitochondrial sequence data (10,112 bp) obtained from GenBank/DDBJ (DNA Data Bank of Japan). The results are illustrated in Figures 1, 2, 3, along with the respective accession numbers.

To calibrate the phylogenetic tree, I utilized a total of 19 mammalian fossil calibration ages, following the approach outlined by Benton et al. (2015). Additionally, I integrated one geological event age based on Osozawa et al. (2012). These particular calibration ages employed are detailed in Figures 1, 2, 3 and Table 1.

**TABLE 1:** Fossil calibrations.

Calibration point	Fossil	Family	Infraorder	Order	Ingroup clade	Formation	System	Stage	tMRCA (ma)	Method	Paleontological reference	Geological reference	Benton et al. (2015)	Maximum age (ma) = parent node age (mostly)	Calibration point
A	*Pan troglodytes*	Hominidae	Simiiformes	Primates	*Pan* spp.	Kapthurin Formation	Quaternary	Pleistocene Chibanian	0.545 ± 0.003	Ar–Ar dating	McBrearty and Jablonski (2005)	Deino and McBrearty (2002)	Lacking	> 5.31 ± 0.03 *Orrorin* = X	A
X (not assigned)	*Orrorin* ^a^	Hominidae	Simiiformes	Primates	*Homo* spp.	Lukeino Formation	Quaternary	Pliocene Zanclean	>5.31 ± 0.03	Correlation	Deino and McBrearty (2002)	Deino and McBrearty (2002)	Chimpanzee‐Human (87)	8.072 ± 0.8 *Sivapithecus* = B	X (not asigned)
B	*Chororapithecus* ^a^	Hominidae	Simiiformes	Primates	*Gorilla*	Chorora Formation	Neogene	Miocene Tortonian	8.072 ± 0.8	Ar–Ar dating	Katoh et al. (2016)	Katoh et al. (2016)	Lacking	12.72 ± 1 *Chororapithecus* = C	B
C	*Sivapithecus* ^a^	Hominidae	Simiiformes	Primates	Hominoidea	Chinji Formation	Neogene	Miocene Serravallian	12.72 ± 1.1	correlation	Kappelman et al. (1991)	Johnson et al. (1985)	Crown Hominoidea (86)	24.93 ± 0.49 *Rukwapithecus* = D	C
D	*Rukwapithecus* ^a^	Catarrhini	Simiiformes	Primates	Catarrhini	Nsungwe Formation	Paleogene	Oligocene Chattian	24.93 ± 0.49	U–Pb dating	Roberts, O'Connor, et al. (2010), Roberts, Stevens, et al. (2010)	Roberts, O'Connor, et al. (2010), Roberts, Stevens, et al. (2010)	Crown Catarrhini (84)	33.9 (base of Oligocene) *Oligopithecus*	D
E	*Altiatlasius* ^a^	Lemuriformes	Simiiformes	Primates	Primates	Adrar Mgorn 1	Paleogene	Paleocenen Thanetian	57.6 ± 1.6	Biostratigraphy	Sige et al. (1990)	Dragastan and Herbig (2007)	Crown Primates (81)	63.8 ± 2.2 = F 66.0 (K‐Pg boundary)	E
F	*Paromomys* ^a^	Paromomyidae	/	Plesiadapiformes	Euarchonta	Tullock Formation	Paleogene	Paleogene Danian	63.8 ± 2.2	Ar–Ar dating	Clemen and Wilson (2009)	Clemen and Wilson (2009)	Crown Euarchonta (74)	160.7 ± 0.4 = R 66.0 (K–Pg boundary)	F
G	Leporidae fossil^a^	Leporidae	/	Lagomorpha	Lagomorpha	Cambay Shale	Paleogene	Paleoegne Ypresian	51.96 ± 4.6	Biostratigraphy	Rose et al. (2008)	Rose et al. (2008)	Crown Lagomorpha (76)	57.6 ± 1.6 = H 66.0 (K–Pg boundary)	G
H	*Paramys* ^a^	Ischyromyidae^a^	Protrogomorpha^a^	Rodentia	Rodentia	Fort Union Formation	Paleogene	Paleoegne Thanetian	57.6 ± 1.6	Correlation	Anemone and Dirks (2009)	Anemone and Dirks (2009)	Crown Rodentia (77)	63.8 ± 2.2 = F 66.0 (K–Pg boundary)	H
H1 (not applied)	*Mimotona* ^a^	Mimotonidae^a^	/	Rodentia	Rodentia	Wanghudun Formation	Paleogene	Paleoegne Thanetian	57.6 ± 1.6	Lacking	Dashzeveg and Russell (1988)	Lacking	Crown Glires (75)	63.8 ± 2.2 = F 66.0 (K–Pg boundary)	H1 (not applied)
I	*Karnimata* ^a^	Muridae	/	Rodentia	Muridae (stem)	Dhok Pathan Formation	Neogene	Miocene Tortonian	10.4 ± 0.1	Correlation	Jacobs (1978)	Barry et al. (2002)	Divergence of Mus from Rattus (79)	23.03 (base of Miocene)	I
J	*Hesperocyon* ^a^	Canidae	/	Carnivora	Carnivora	Cypress Hills Formation	Paleogene	Eocene Bartonian	38.8 ± 1.6	Correlation	Robinson et al. (2004)	Robinson et al. (2004)	Crown Carnivora (69)	51.25 ± 0.31 = K 66.0 (K–Pg boundary)	J
K	*Orohippus* ^a^	Equidae	/	Perissodactyla	Equidae (stem)	Green River Formation	Paleogene	Paleoegne Ypresian	51.25 ± 0.31	Ar–Ar dating	Grande (1980)	Smith et al. (2003)	Lacking	63.8 ± 2.2 = N	K
L	*Eotragus* ^a^	Bovidae	/	Cetartiodactyla	Bovidae (stem)	Vihowa Formation	Paleogene	Miocene Burdigalian	18.205 ± 2.235	Correlation	Solounias et al. (1995)	Bibi (2013)	Crown Bovidae (73)	25.425 ± 2.395	L
M	*Himalayacetus* ^a^	Pakicetidae^a^	/	Cetacea^a^	Cetartiodactyla	Subathu Formation	Paleogene	Paleoegne Ypresian	53.25 ± 0.75	Biostratigraphy	Bajpai and Gingerich (1998)	Bajpai and Gingerich (1998)	Crown Artiodactyla (70)	63.8 ± 2.2 = N 66.0 (K–Pg boundary)	M
N	*Adunator* ^a^	Erinaceidae	/	Eulipotyphla	Eulipotyphla (stem)	Fort Union Formation	Paleogene	Paleoegne Danian	63.8 ± 2.2	Correlation	Bown and Schankler (1982)	Bown and Schankler (1982)	Crown Eulipotyphla (68)	160.7 ± 0.4 = R	N
O	*Eritherium* ^a^	/	/	Proboscidea	Afrotheria	Sidi Chennane quarries	Paleogene	Paleoegne Thanetian	57.6 ± 1.6	Biostratigraphy	Gheerbrant (2009)	Gheerbrant (2009)	Crown Afrotheria (63)	160.7 ± 0.4 = R	O
P	*Riostegotherium* ^a^	Dasypodidae	/	Cingulata	Xenarthra	Itaboraí Formation	Paleogene	Paleoegne Thanetian	57.6 ± 1.6	Correlation	Scillato‐Yane (1976)	Woodburne et al. (2014)	Crown Xenarthra (62)	160.7 ± 0.4 = R	P
Q	*Djarthia* ^a^	/	/	/	Marsupialia	Tingamarra Murgon	Paleogene	Paleoegne Thanetian	57.6 ± 1.6	Correlation	Beck et al. (2008)	Beck et al. (2008)	Crown Marsupialia (58)	130.7 ± 1.4 (*Sinodelphys* Jehol Biota^a^)	Q
R	*Juramaia* ^a^	/	/	/	Theria (stem)	Tiaojishan Formation	Jurassic	Oxfordian	160.7 ± 0.4	Ar–Ar dating	Luo et al. (2011)	Luo et al. (2011)	Crown Theria (59)	162.7 ± 1.1 = S	R
S	*Ambondro* ^a^	/	/	/	Prototheria (stem)	Isalo Group	Jurassic	Bathonian	162.7 ± 1.1	Correlation	Flynn et al. (1999)	Flynn et al. (1999)	Crown Mammalia (57)	201.3 ± 0.2 (base of Jurassic)	S
Z	geological event	Mustelidae	/	Carnivora	*Lutra* sp.	Ryukyu	Quaternary	Pleistocene Calabrian	1.55 ± 0.15	Biostratigraphy	Osozawa, Kanai, et al. (2021)	Osozawa et al. (2012)	/	/	X

*Note*: Z, geological event calibration. See Figures 1 and 2 for fossil calibration points as applied.

^a^ Extinct.

Many authors, as listed in Table 2, have constructed timetrees for mammals, focusing on the ordinal radiation after, across, or before the Cretaceous‐Paleogene (K–Pg) boundary, aligning with the mass extinction event of non‐avian dinosaurs (Bininda‐Emond et al., 2007; Carretero et al., 2022; dos Reis et al., 2012, 2018; Liu et al., 2017; Meredith et al., 2011; Murphy et al., 2021; O'Leary et al., 2013; Pozzi et al., 2014; Springer et al., 2019; Upham et al., 2019, 2021). Alternatively, Nishihara et al. (2009) and Foley et al. (2023) linked superordinal differentiation to the middle Cretaceous supercontinent breakup, while Liu et al. (2017) associated it with the Cretaceous angiosperm radiation identified by Magallon et al. (2015). By integrating multiple time calibration points from the geologic record, I am now able to reliably date the mammalian tree (Figures 1, 2, 3), and establish correlations between biological divergences and global geological events.

**TABLE 2:** List of representative dated trees.

References	Target	Tree siza	Genome size	Application	Reference	Calibration point	K–Pg diversification model
Bininda‐Emond et al. (2007)	Mamalian	4510	51,089	(PAUP* v4.0b10)	Swofford (2002)	30	Short fuse
Meredith et al. (2011)	Mamalian	164	58,382	(PAUP* v4.0b10)	Swofford (2002)	31	Short fuse
dos Reis et al. (2012)	Mamalian	274	20,600,000	MCMCTree in PAML (4.4e)	Yang (2007)	26	Long fuse
Bibi (2013)	Bovidae	127	ca. 14,000	BEAST 1.7.4	Drummond et al. (2012)	16	Explosive
O'Leary et al. (2013)	Placentalia	86	4541 phenomic characters	MrBayes 3.2.1	Ronquist and Huelsenbeck (2003)	82	Explosive
Pozzi et al. (2014)	Primates	62	14,043	MrBayes 3.2.1	Ronquist and Huelsenbeck (2003)	17	Short fuse
Foley et al. (2016)	Placentalia	286	100,000	MCMCTree in PAML (4.9e)	Yang (2007)	99	Long fuse
Phillips (2016) = Meredith et al. (2011)	Placentalia	164	58,382	MCMCTree in PAML (4.9e)	Yang (2007)	31	Short fuse
Liu et al. (2017)	Placentalia	90	4388 gene dataset	MCMCTree in PAML (4.9e)	Yang (2007)	21	Long fuse
Wu et al. (2017)	Placentalia	89	1185 gene dataset	MCMCTree in PAML (4.8)	Yang (2007)	30	Long fuse
dos Reis et al. (2018)	Primates	372	3,441,106	MCMCTree in PAML (4.9e)	Yang (2007)	25	Explosive
Springer et al. (2019) = O'Leary et al. (2013)	Placentalia	274	20,600,000	MCMCTree in PAML (4.4e)	Yang (2007)	26	Explosive
Upham et al. (2019)	Mamalian	4098	39,099	MrBayes 3.2.7	Ronquist et al. (2012)	17	Long fuse
Upham et al. (2021)	Mamalian	5911	39,099	MrBayes 3.2.7	Ronquist et al. (2012)	17	Long fuse
Murphy et al. (2021)	Placentalia	286	100,000	MCMCTree in PAML (4.9e)	Yang (2007)	99	Long fuse
Hassanin et al. (2021)	Carnivora	220	14,892	BEAST v2.4.7	Bouckaert et al. (2014)	22	Explosive
Carretero et al. (2022)	Mamalian	4705	51,093–389,827	MCMCTree in PAML (4.9e)	Yang (2007)	92	Long fuse
Foley et al. (2023)	Placentalia	241	411,110	MCMCTree in PAML (4.9e)	Yang (2007)	23	Long fuse
Present paper	Mamalian	133	10,112	MEGA 11	Tamura et al. (2021)	20	Explosive
Present paper	Mamalian	133	10,112	BEAST v1.10.4	Suchard et al. (2018)	20	Explosive

*Note*: A large tree size, genome size, and an abundance of calibration points may aid in resolving dated phylogenies, but the current robust calibration enhances both dating and phylogenetic accuracy.

The primary novelty of this paper lies less in resolving dated phylogenies, but more in the methodological approach of correlating base substitution rates with time. Time‐dependent substitution rates were previously reported by Ho et al. (2005), Papadopoulo et al. (2010), and in the latest versions by Osozawa and Wakabayashi (2022, updated in 2023).

The resulting timetree for mammals was generated using the BEAST v1.10.4 platform and visualized using FigTree v1.4.4 (Suchard et al., 2018). I assessed the base substitution rate over time by examining the rate depicted in FigTree (refer to the end of the Methods section). This rate is not strictly constant, given the use of a relaxed clock model. To illustrate this, I created a diagram (inset in Figures 2 and 3) that illustrates the relationship between the base substitution rate and the age of nodes. The diagram indicated that the rate did not remain constant or randomly variable but instead exhibited an exponential increase toward recent times. This pattern is reflective of mammalian radiation and is not directly linked to the K–Pg mass extinction event.

## 2 METHODS

Molecular‐clock analyses have been extensively reviewed by Ronquist et al. (2012) and Ho and Duchene (2014). To establish a geologically dated tree, the RelTime method (Tamura et al., 2021) implemented in MEGA 11, as well as Bayesian Markov Chain Monte Carlo (MCMC) methods like MCMCTree in PAML (4.9e 2017; Yang, 2007), BEAST v1.10.4 (Suchard et al., 2018), and BEAST 2 (v2.7 2023; Bouckaert et al., 2019) were employed. The total‐evidence dating calibration function (Zhang et al., 2016) was also utilized in BEAST v2.7 and MrBayes (v3.2.72019; Ronquist et al., 2012).

For this study, I specifically chose the latest version of MEGA 11 (Tamura et al., 2021) and BEAST v1.10.4 (Suchard et al., 2018) due to their well‐designed time calibration function and established protocols for clock analyses.

### 2.1 Application of whole mitochondrial sequence data

To analyze the mammalian tree, I utilized whole mitochondrial sequence data that were previously uploaded and analyzed by Pozzi et al. (2014) for most primates and representative mammals. The accession numbers for these sequences can be found in their Table 1, as well as in Figures 1 and 2 of my study. In addition, I expanded my dataset by including representative mammalian data from GenBank/DDBJ, encompassing every order (refer to Section 1). These data were incorporated into the present maximum parsimony and Bayesian inference analyses.

I integrated whole mitochondrial sequence data from fossil species as well. The GenBank/DDBJ accession numbers for these sequences can be found in Figures 1 and 2, along with the corresponding references in GenBank/DDBJ. The included fossils were *Homo sapiens* ssp. *Denisova*, *Homo sapiens neanderthalensis*, *Mammuthus primigenius*, and an extinct Japanese otter species, *Lutra lutra* (Waku et al., 2016). These subfossil data were acquired through meticulous procedures, including clean bench protocols, Illumina sequencing, Oxford Nanopore sequencing, and other appropriate methods. Additionally, I incorporated data from the endangered Tasmanian devil (*Sarcophilus harrisii*).

Phylogenetic research employing complete mitochondrial data has experienced notable advancements, enabling the construction of highly resolved and accurate phylogenetic trees (Bi et al., 2023; Łukasik et al., 2018; Yuan et al., 2022; Zhang et al., 2021).

The whole mitochondrial sequences were aligned using Clustal W in MEGA 11. The alignment process involved opening the sequence data in the main window by selecting Data > Open a File/Session. Subsequently, in the Alignment Explorer window, Alignment > W Align by ClustalW was selected. Gap regions present in nonprotein coding tRNAs, rRNAs, and D‐loop regions were excluded from the aligned sequences. The resulting alignment encompassed protein coding sequence data amounting to 10,112 bp (e.g., the protein coding genes accounted for 12,774 bp in the mitochondrial genome of AJ421452; the length may vary among GenBank/DDBJ data).

It is worth noting that my analysis using whole mitochondrial sequences is comparable to genome analyses involving concatenated genes of 39,099 bp (refer to table 2 in Upham et al., 2019). In my specific analysis, I did not find it necessary to perform concatenation of genes using SeaView (Gouy et al., 2010) and partitioning using PartitionFinder 2 (Lanfear et al., 2016) found in MCMCTree (Yang, 2007), BEAST v1.7 (Drummond et al., 2012), BEAST v2.5 to v2.7 (Bouckaert et al., 2014, 2019), and MrBayes (Ronquist et al., 2012; Upham et al., 2019 for the concatenation of the 31 gene fragments and partitioning). Therefore, the 10,112 bp sequence represents a single long partition in the present analyses conducted using MEGA 11 and BEAST v1.10.4.

It is important to recognize that mitochondrial genes tend to exhibit higher resolution and a slightly higher base substitution rate compared to nuclear genes, particularly for ancient ages extending up to the Jurassic period (referring to Figures 1, 2, 3). It is noteworthy that mitochondrial genes have not reached the saturation point of mutation (Osozawa et al., 2012; Osozawa, Sato, et al., 2017; Osozawa & Wakabayashi, 2022; see end of Section 2 and Section 3). Moreover, I disregarded paralogs present in nuclear genes, following the functional genomics outlined by Gabaldon and Koonin (2013).

### 2.2 Maximum parsimony analyses by MEGA 11: Tutorial

A parsimony inference (PI) tree was constructed using the software MEGA 11, following the protocol described in Tamura et al. (2021). For a detailed tutorial, you can refer to the MEGA 11 website.

To construct a parsimony tree in MEGA 11, you can treat the whole mitochondrial gene and unrelated sequences separately, although the concatenation utility is available. To concatenate sequence alignments, click DATA > Concatenate Sequence Alignments in the main MEGA window. Then, select a folder containing the fasta‐formatted sequence data. It is important to check the concatenated data by realigning it using the aforementioned MEGA 11 function.

MEGA 11 provides five statistical methods for tree construction, and in this case, I draw a maximum parsimony tree. To construct a maximum parsimony tree, click Phylogeny > Construct/Test Maximum Parsimony Tree(s). Input the file containing the whole mitochondrial data and run the analysis. Check the topology of the output parsimony tree to ensure it is reasonable.

To proceed with the RelTime dating analysis (Tamura et al., 2021), the current tree should be exported as a Newick file (.nwk). To export the current tree in Newick format, click File > Export Current Tree (Newick).

For the RelTime analysis, click CLOCKS > Compute Timetree > RelTime‐ML. The Timetree Wizard will appear. Click “Browse” to input the whole mitochondrial sequence data and load the tree file (the .nwk file) created from the parsimony tree. To specify the outgroup, click “Select Branch” and check the Monotremata‐Metatheria node. Click “OK.” To calibrate the nodes, click “Add Constraints” and refer to Figure 1 (or the provided information) for the calibration node dates, which are common to the BEAST v1.10.4 calibration node dates. Note that in MEGA 11, as well as in BEAST v1.10.4, multiple points of calibration can be simultaneously applied.

### 2.3 Phylogenetic analyses by BEAST v1.10.4: Tutorial

The protocol has evolved with the newer versions of BEAST v1.8 (and v1.10.4) from BEAST v1.7 (Drummond et al., 2012). It is recommended to use the latest version of BEAST v1.10.4. For a detailed tutorial, explanation, and terminology of the BEAST software, you can refer to Heath (2022).

To construct the Bayesian inference (BI) tree (Figures 2 and 3), the BEAST software package was used. The analysis involves running BEAUti v1.10.4, BEAST, TreeAnnotator v1.10.4, and FigTree v1.4.4, in that order. It is important to download the BEAGLE Library before using the BEAST software platform. Tracer v1.6 was utilized to check the calculation status and estimate the mean base substitution rate.

For a graphical explanation and detailed settings in BEAUti, you can refer to Osozawa (2021). Additional references that employed BEAST v1.10.4 are Osozawa, Kanai, et al. (2021) and Osozawa and Wakabayashi (2022).

In BEAUti, the following software settings were used:

Partitions: The fasta files were loaded using the “Import Data” or plus button. The whole mitochondrial sequence data of 10,112 bp was defined as a single partition, appearing in the Partition box.
Taxa: The taxa were added as an ingroup by selecting the plus button in BEAUti. In total, my analysis included 133 species from 56 families and 23 orders. It is important to note that the size of the tree (i.e., the number of species) does not directly impact fossil age estimation, calibration, and dating purposes. According to Upham et al. (2019), a maximum tree size of approximately 800 species is possible for running MrBayes within a few weeks (refer to their table 4). They merged their total 4098 extant species tree (shown in their figure 1) from these delimited trees. In the left screen of BEAUti, the Taxon Set was configured with monophyletic boxes checked for all taxa, while the stem box was checked on a case‐by‐case basis (as outlined in Table 1). The right screen displayed the included monophyletic taxa, representing specific clades. For the input in Figures 2 and 3, calibration dates (see below) were set in the Priors section of BEAUti.
Tips: Default (Osozawa & Wakabayashi, 2022; Osozawa, Kanai, et al., 2021; https://beast.community/first_tutorial). The Tips panel allows for assigning dates to extant taxa. However, taxon dates (or “tip dates”) are only important in certain cases, for example, when they sampled from fast evolving viruses or subfossil ancient DNA material (c.f., King & Rücklin, 2020). In the present case, I am analyzing a tree that represents millions of years of distinct evolution, and the dates of the tips can be assumed to be zero. For extinct taxa such as *Homo sapiens* ssp. *Denisova*, *Homo sapiens neanderthalensis*, *Mammuthus primigenius*, and the extinct Japanese *Lutra lutra*, their tip dates can be considered close to 0 Ma, although there may be studies attributing specific dates, such as 0.04 Ma for *Homo sapiens neanderthalensis* (Higham et al., 2014). Tips should the default, that is, the contemporary sampled taxa all have a date of zero and I do not select the “Use tip dates” box. Tip dating function in MEGA 11 is similarly not recommended to employ in a case of concerning geological time scale. Note that the current tip dating method differs from the total‐evidence tip dating.
Sites: Substitution Model: HKY (Hasegawa, Kishino and Yano) model, Base frequencies: Empirical, Site Heterogeniety Model: Gamma, Number of Gamma Categories: 4, Partition into codon positions: Off. The GTR model generates similar topology. The same option is found in Set Options; Timetree Wizard; MEGA 11.
Clocks: Clock Type: Uncorrected relaxed clock, Relaxed Distribution: Lognormal. Uncorrelated relaxed clocks allow each branch of a phylogenetic tree to have its own evolutionary rate under log‐normal distribution, and the node rate is the rate median of three branches (Drummond et al., 2006). RelTime method in MEGA 11 follows relaxed clock method.
Trees: Tree Prior: Speciation: Yule Process. See details in Heath (2022).
Priors: tmrca (time of MRCA; time of most recent common ancestor) was input from the calibration point date noted below as Prior Distribution: Normal, and as the Mean and Standard deviation. In MEGA 11, also normal distribution was selected.
MCMC: Length of chain: 10,000,000. Increased length such as 20,000,000 only spends time and unaffected on output.

Running BEAST was done by incorporating xml input file made by BEAUti. The tree files were input into TreeAnnotator, and the consequent tree was drawn by FigTree v1.4.4. Note that the same setting in BEAUti does not produce the completely same output in every run reflecting Bayesian MCMC method (non‐reproducible in some case).

### 2.4 Non‐utilization of BEAST 2 and MrBayes

Considering relevant factors and the specific requirements of the present study, I utilize MEGA 11 and BEAST v1.10.4 instead BEAST 2 and command line MrBayes.

My approach deviated from previous studies, such as Osozawa, Ogino, et al. (2016), Price et al. (2019), and Hill et al. (2021), which employed BEAST v2.5 (Bouckaert et al., 2014; v2.4 named *BEAST = Star BEAST; Heled & Drummond, 2010). Instead, I chose to use BEAST v1.8 and subsequently v1.10.4. While the calibration function in BEAUti of BEAST v2.5 bears similarities to BEAST v1.10.4, there are notable differences. In BEAST v2.5, the “Partition” tab only permits the input of individual DNA sequence data. Consequently, if the sequence data are not concatenated, separate BEAST runs must be conducted for each set of applied sequence data (e.g., mitochondrial COI and nuclear 16S rRNA), as demonstrated by Osozawa, Fukuda, et al. (2016) and Osozawa, Ogino, et al. (2016). The resulting tree files from these runs must then be combined into a single file using LogCombiner. However, when merging these tree files, the branches in the resultant tree become folded, reflecting the incongruent topology arising from different sequence data sources, such as mitochondrial COI and nuclear 16S rRNA. To mitigate this issue, Osozawa, Fukuda, et al. (2016) and Osozawa, Ogino, et al. (2016) employed DensiTree, packaged in the present BEAST v.2.7.5, to obscure the folds. Consequently, I discourage the use of the older version, BEAST v2.5, due to the inconvenience and potential confusion caused by folded branches in the combined tree.

In the case of BEAST v2.6, released in May 2019, and BEAST v2.7, released in 2023 (Bouckaert et al., 2019), node dating by inputting tMRCA was introduced, a feature inherited from BEAST v2.5. However, setting the dates is more complicated than in BEAST v1.10.4. A tutorial for these newer versions can be found in Barido‐Sottani et al. (2018) and Ogilvie (2020). These versions saw significant protocol changes, notably allowing the inclusion of morphological data alongside molecular data through the implementation of total‐evidence dating (Zhang et al., 2016). For extinct species, total‐evidence dating is set at their youngest fossil age, while for extant species, it is set at zero age. However, the youngest fossil age is often poorly constrained.

In the context of MrBayes, as well as in BEAST v2.6 and v2.7, it is important to clarify that the term “tip” in total‐evidence dating (Ronquist et al., 2019; Upham et al., 2019) does not refer to terminal nodes for extant species. Instead, it refers to the tip node representing extinct fossil species from ancient times. The tip date for fossil species is inferred from the fossil age, and it is worth noting that the age assigned is not necessarily the minimum age for the oldest fossil, but rather the youngest fossil, which is often poorly constrained. Additionally, it is crucial to ensure that these fossil species are indeed extinct, and determining their relative placement in relation to the lineage of extant species can be problematic, as it involves the concept of ghost lineage (as discussed in O'Leary et al., 2013; Springer et al., 2019). It is important to understand that this tip dating (= total‐ evidence dating) does not contribute to the quality of node dating (c.f., Upham et al., 2019). Nevertheless, the use of total‐evidence analyses with MrBayes has become increasingly common in recent studies (e.g., Coiro et al., 2023; Troyer et al., 2022; Zhang & Wang, 2019).

### 2.5 Fossil calibration protocols BEAST v1.10.4 and MCMCTree

For estimating evolutionary rate, calibration is essential, and fossil and geological event calibration is possible (Ho & Duchene, 2014). A node calibration is done using the oldest recognized representative fossil of a specific clade, and this fossil is used to constrain its “minimum age” (Donoghue & Yang, 2016; Ronquist et al., 2012). Due to the fragmentary nature of the fossil record and the relying on the oldest fossil, the exact MRCA of a clade may never be found.

For the mammalian fossil calibration in the command line software MCMCTree in PAML (4.9e); however, both the minimum and maximum age constraint for a specific node has been used (Benton & Donoghue, 2007). The minimum age is expected to be the oldest fossil age younger than the specific branch node within the clade. The maximum age is inferred by an additional fossil age older than that specific node, that is, the stem age is representative, although the definition is sometimes unclear and controversial. Minimum ages are generally more accurate and reliable than maximum ages (Hassanin et al., 2021). In actual MCMCTree analyses (e.g., Carretero et al., 2022; dos Reis et al., 2012, 2018), a specific node age is estimated by prior divergence time applying minimum and maximum ages, although the maximum age is only roughly estimated. A root age for the tree, which is the oldest maximum constraint from a third fossil age, is required, in order for the algorithm to converge on a unique solution in MCMCTree.

The calibration protocol of BEAST v1.10.4, v1.8, and MEGA 11 is to place a time of the most recent common ancestor (tMRCA) of the ingroup extant taxa (= a crown node age of a specific clade or monophylum), and I can approximate the tMRCA with the oldest fossil age known at the time of analyses. The minimum ages proposed by Benton et al. (2015) for MCMCTree equivalent to the tMRCA for BEAST v1.10.4, and a maximum age is not needed to constrain calibration in BEAST v1.10.4. A root age in the MCMCTree analyses can correspond to the oldest tMRCA of all the ingroups or clades in BEAST v1.10.4, and a third fossil age is not needed (the root age is automatically estimated from the remaining younger tMRCAs). Note again that the BEAST v1.10.4 (v1. 8) protocol has been significantly revised from BEAST v1.7 (Drummond et al., 2012) that shared a similar calibration protocol with MCMCTree.

Both the BEAST v1.10.4 and MCMCTree input prior node age and then an output posterior node age are expected to be coincident. The difference is that the BEAST v1.10.4 node age (tMRCA; input mean and standard deviation; see next section) is younger than MCMCTree node age, if defined as the mean: (maximum age + minimum age)/2 with standard deviation: (maximum age – minimum age)/2, so that a timetree created by MCMCTree may be shifted to older dates relative to that generated by BEAST v1.10.4. The difference may stem from the calibration protocol, specifically the consideration of the maximum age. In the results section, I tested the effect of applying tMRCA as the minimum age or (maximum age + minimum age)/2. Phillips (2016) emphasized that tight calibration across the tree is vital to buffer against rate model errors and this must include allowing maximum ages to be tight when robust fossil records permit this.

I employed normal prior distribution in BEAST v1.10.4, but selection of lognormal distribution may impact node dating, and so I compared results generated by these two prior distribution settings.

I noted above which is the oldest relevant fossil within the clade is in question. For my analysis, I assume that tMRCA equals the oldest known representative fossil age of the specific clade. The present timetree was made by robust calibration by a total of 20 points ranging in age from 0.545 Ma up to 162.7 Ma, and the dated tree is balanced or equilibrated (if not, some branches would be folded). The run time for BEAST exceeded 5 hours when analyzing the whole mitochondrial long sequence data in my study. It is worth noting that for longer sequence data, such as genome‐scale data, the run time can be even longer (Upham et al., 2019, their table 4). In order to develop the final dated mammalian timetree, I conducted dozens of runs to ensure robustness and accuracy in my results. Contrastingly, the run time for RelTime analysis is only 10 min.

Note that multiple calibration points are particularly helpful in relaxed‐clock methods where the rate is allowed to vary among branches in the tree; multiple calibrations throughout the tree act as anchor points, allowing the method to estimate the patterns and degree of rate variation more accurately, that is, the interaction of multiple dates works better to constrain the overall timing of the whole tree. Good estimates of rate variation are required from the well‐calibrated regions of the tree so that the pattern can be extrapolated to other parts of the tree that are poorly calibrated (Phillips, 2016). For such analyses, the maximum age in MCMCTree can be replaced by the age of lower node at a step from the specific node with tMRCA in BEAST v1.10.4.

The fossil age should be chronologically checked strictly following the protocol proposed by Parham et al. (2012) that is geologically robust. Reliable radiometric age by Ar–Ar or U–Pb radiometric dating is recommended. An example of such a robust age is represented by the 98.79 ± 0.62 Ma age of the fossiliferous Burmese amber (Shi et al., 2012; that do not contain mammalian fossils, however). In some cases a fossil age has been determined by biostratigraphy rather than direct radiometric dating so it is represented as a chronostratigraphic unit from the Geologic Time Scale such as Cenomanian (97.2 ± 3.3 Ma). Ages for such chronostratigraphic horizons used herein are from International Chronostratigraphic Chart by International Commission on Stratigraphy (Cohen et al., 2013; using v2022/10 updates).

According to Klopfstein (2021), recent node dating approaches for insects including those of Misof et al. (2014) and Montagna et al. (2019) have revealed a problem: different studies using the same molecular data and even the same sets of fossils commonly arrive at drastically different age estimates. A major reason for these differences is well known: even well‐dated and firmly placed fossils can only provide a minimum age for a particular node (c.f., Murphy et al., 2021). This further justifies my use of minimum ages for generating my timetree, because the maximum age affected on dating inflated (Klopfstein, 2021). As a precedent, Bibi (2013) generated a Bovidae dated tree considering only minimum ages.

### 2.6 Actual practice of fossil calibration (Table 1)

Specific calibrations used in this study were input as described below for the points of prior (input) ages (tMRCAs) (shown on Figures 1, 2, 3 as green stars), and these dates were input in “CONSTRAINTS” in Calibration Editor, MEGA 11 or “Priors” in BEAUti as mean and standard deviation (For example for a date such as 98.79 ± 0.62 Ma, mean = 98.79, standard deviation = 0.62). Corresponding ingroup species (clade; monophylum) were included in ingroup taxa by “Taxon Set” on the “Taxa” screen in BEAUti. This function is lacking in MEGA 11, so once specified clade in Calibration Editor screen cannot be modified.

Figures S1 and S2 were made by prior (input) age of mean: (maximum age + minimum age)/2 and standard deviation: (maximum age – minimum age)/2, in the similar fashion to MCMCTree for testing the effect of using maximum ages (see previous section). Note, however, that MCMCTree dating is statistically much more complex. Figure S3 was made by prior lognormal distribution whereas Figures S1 and S2 made using normal distribution (see previous section).

Ingroup species or clade species were defined on the basis as to whether they are monophyletic (a function of BEAUti), although a clade must be obviously monophyletic. For example, this analysis is used to determine whether various Afrotheria species should be included in the Afrotheria clade. For convenience in selecting ingroup taxa, I entered headings such as “Afro” in the sequence file. I checked stem age (a function of BEAUti) for five ingroup species (Prototheria, Theria, Bovidae, Eulipotyphla, and Equidae), because the fossil age is not defined to represent tMRCA (within the clade) but its stem (Figures 2 and 3; Table 1). These functions are lacking in MEGA 11.

My fossil calibration points totaled 19, and covered a time range from 0.545 to 162.7 Ma (root age; 162.7 Ma; Table 1; two points in Table 1 were not applied). In each case, I evaluated the original references and performed additional literature research to find additional chronologic and geologic supporting evidence. If I deemed it necessary, I modified the age from the original reference. In Figures 1, 2, 3, I showed fossil calibration points from A to S with the prior input age, but the calibration point Z is a geological event calibration at 1.55 ± 0.15 Ma noted below.

Benton and Donoghue (2007) and Benton et al. (2015) reviewed many calibration data available for animals, and the following mammalian calibration points and dates are addressed by their proposal, except for calibration point X, that cannot be applied, and K that I suggest. These data were for MCMCTree analyses, and the maximum ages by Benton et al. (2015) were also addressed below (also in Table 1) to construct Figures S1 and S2. In Figure 3 and Figure S2, fossils to adjust minimum ages (times of MCRA) were also shown (Benton et al., 2015).

Calibration point A: Fossil chimpanzee was reported from the East African Rift zone (McBrearty & Jablonski, 2005), and dated by the Ar–Ar method as 0.545 ± 0.003 Ma (Deino & McBrearty, 2002). The time of MRCA is a node date between two *Pan* species (Figures 1 and 2), although Benton et al. (2015) cited the above reference only and did not address the chimpanzee split (point X). I here set the maximum age at >5.31 ± 0.03 Ma of the calibration point X based on considerations presented below.

Calibration point X: Crown Hominini (87) by Benton et al. (2015); Not assigned in this paper. Benton and Donoghue (2007) and Benton et al. (2015) noted that the Lukeino Formation of Kenya is the source of *Orrorin* (Hominina; dated at 6.56–5.73 m.y. from Ar/Ar dates on volcanic layers; Deino & McBrearty, 2002). However, the Lukeino Formation at Kapcherberek, including the fauna, was deposited during chron C3r and can thus be constrained to the interval 5.88–5.72 Ma according to Deino and McBrearty (2002), but this correlation to the magnetic polarity timescale is uncertain leaving the age of this formation somewhat problematic. Ar/Ar dates for ignimbrite and associated air‐fall tuff of the basal Chemeron Formation directly overlying the Lukeino Formation are 5.31 ± 0.03 Ma (Deino & McBrearty, 2002). The best age constraint for the *Orrorin* locality is that it is older than 5.31 ± 0.03 Ma (apparently reliable ages from the oldest overlying strata). The proposed lower (maximum) age limit of 6.56 Ma by Benton and Donoghue (2007) is rendered questionable by apparent conflict between the Ar–Ar dates and magnetostratigraphy reviewed above. Benton and Donoghue (2007) and Benton et al. (2015) noted that dating of the *Sahelanthropus* (oldest Hominina) beds in Chad is not direct. Biostratigraphic evidence from mammals in particular, but with cross‐checking from fish and reptile specimens, only indicates that the unit is late Miocene (i.e., 5.33 to ca.11.6 Ma), and it is older than the Lukeino Formation of Kenya (thus older than 5.31 ± 0.03 Ma), and may be equivalent (biostratigraphically correlated) to the lower fossiliferous units of the Nawata Formation of Kenya (dated by traditional K‐Ar method by diverse and perhaps dubious ages of 7.4 and 6.5 Ma; Vignaud et al., 2002). The crown Hominini age is thus uncertain or >5.31 ± 0.03 Ma, and I did not adopt point X for my calibration or prior setting. I attempted to estimate node X age from the other reliable calibration data. The maximum age may be defined for *Chororapithecus* as 8.072 ± 0.8 Ma in calibration point B below, but I did not use this as a maximum age in my analysis.

Calibration point B: Fossil gorilla (*Chororapithecus abyssinicus*) was reported from the East African Rift region, and reliably dated by concordant Ar–Ar dates and magneto‐stratigraphy, as 8.072 ± 0.8 Ma (Katoh et al., 2016). The time of MRCA is a node date of Homininae (*Gorilla*, *Pan*, and *Homo*). I set maximum age at 12.72 ± 1.1 Ma for *Sivapithecus* in calibration point C below.

Calibration point C: Crown Hominoidea (86) by Benton et al. (2015). *Sivapithecus* (Ponginae) fossil was found in strata from Siwalik, Pakistan (Kappelman et al., 1991) that is considered to be the Serravallian stage of the Miocene epoch (12.72 ± 1.1 Ma; Johnson et al., 1985). Benton et al. (2015) proposed the minimum age of 11.62 (12.72–1.1) Ma, and maximum age as a stem date of fossil Ponginae at the base of the Oligocene (33.9 Ma). However, the maximum age at 33.9 Ma should be replaced by younger node D age of 24.93 ± 0.49 Ma.

Calibration point D: Crown Catarrhini (Cercopithecoidea; Old World monkeys + Hylobatidae + Hominoidea) (84) by Benton et al. (2015). The Nsungwe Formation, Tanzania, contains *Rukwapithecus*, and yields a U–Pb age is 24.93 ± 0.49 Ma (Roberts, O'Connor, et al., 2010; Roberts, Stevens, et al., 2010; Stevens et al., 2013). Benton et al. (2015) proposed the minimum age of 24.44 (24.93–0.49) Ma, and maximum age from a fossil Catarrhini at the base of the Oligocene (33.9 Ma).

Calibration point E: Crown Primates (81) by Benton et al. (2015). Fossil *Altiatlasius* (euprimate) was found from Morocco (Sige et al., 1990) from strata considered to have an age of Thanetian (57.6 ± 1.6 Ma; Dragastan & Herbig, 2007). A maximum age at 66.0 Ma is from a Danian primate fossil, but I prefer 63.8 ± 2.2 Ma from node F below.

Calibration point F: Crown Euarchonta (74) by Benton et al. (2015). Fossil *Paromomys* (Primates, Euarchontoglires) was found from Montana, USA in rocks considered to be of the Danian stage (63.8 ± 2.2 Ma; Torrejonian North American Stage; Clemen & Wilson, 2009). The proposed maximum age is the same as tMRCA of calibration point R: Crown Theria (59) by Benton et al. (2015), and *Juramaia sinensis* from strata in Liaoning, Northeastern China with an Ar–Ar age of 160.7 ± 0.4 Ma (Luo et al., 2011). I consider this too loose a time constraint and instead prefer a maximum age of 66.0 Ma from the Danian primate fossil.

Calibration point G: The common ancestor of Lagomorpha (Leporidae; leporids; rabbits and hares + Ochotonidae; ochotonids; pikas) (76) by Benton et al. (2015). The Indian leporid fossil horizon was considered to be Ypresian (51.96 ± 4.6 Ma) based on a correlative foraminifer zone (Rose et al., 2008), and the tMRCA of Lagomorpha equals the stem age of Leporidae at 51.96 ± 4.6 Ma. The proposed maximum age is 66.0 Ma (K–Pg boundary) from a Glires fossil (Benton et al., 2015), but I prefer 57.6 ± 1.6 Ma from node H below as a maximum age.

Calibration point H: Crown Rodentia (77) by Benton et al. (2015). Fossil *Paramys* was found from the upper Fort Union Formation, Bighorn Basin, USA, and considered to be of the Thanetian stage (57.6 ± 1.6 Ma; Anemone & Dirks, 2009). The maximum age proposed was the same as noted above (66.0 Ma), but I prefer 63.8 ± 2.2 Ma at node F. H1: I did not apply Crown Glires (75) by Benton et al. (2015) (77), because age of the Wanghudun Formation with *Mimotona*, China, is uncertain (Dashzeveg & Russell, 1988).

Calibration point I: Intra‐Murinae, Divergence of *Mus* from *Rattus* (79) by Benton et al. (2015). The fossil (*Karnimata*) horizon from Siwalik, Pakistan (Jacobs, 1978), was dated by biostratigraphy, magnetostratigraphy, and oxygen isotope chronology at 10.4 Ma (Barry et al., 2002). The age of fossil *Antemus*, Pakistan (Jacobs, 1977), proposed as a maximum age, is rather loosely constrained, and considered to be 23.03 Ma (base of Miocene).

Calibration point J: Crown Carnivora (69) by Benton et al. (2015). The fossil *Hesperocyon* was from the Canadian Duchesnian (North American land mammal age) estimated at 38.8 ± 1.6 Ma (Bryant, 1993; Robinson et al., 2004). The maximum age proposed is 66.0 Ma (K–Pg boundary) from the Carnivora stem fossil, but I prefer 51.25 ± 0.31 Ma from node K.

Calibration point K: *Orohippus* (Equidae) was reported from the Green River Formation, USA (Grande, 1980). Ar–Ar dating applied to the silicic tuff within the formation yields ages of 53.5–48.5 Ma (weighted average of 51.25 ± 0.31 Ma; Smith et al., 2003). Although *Orohippus* evolved from equids such as *Sifrhippus* and *Eohippus* from the Ypresian Willwood Formation (Froehlich, 2002), I used above date for my calibration for Perissodactyla (stem). Bat (Phyllostomidae) fossils, as well as primate fossils, were also reported from the Green River Formation (Grande, 1980), and bat (not calibrated due to trifurcation) is included in my timetree with the similar output node age (53.26 Ma; Figure 1). The maximum age is 63.8 ± 2.2 Ma at node N.

Calibration point L: Crown Bovidae = Divergence of Bovinae (cow) and Antilopinae (sheep) (73) by Benton et al. (2015). The fossil (*Eotragus*) horizon in the Siwalik Deposits (Solounias et al., 1995) is of the Burdigalian stage (18.205 ± 2.235 Ma) based on the paleomagnetic chronology (Bibi, 2013). The maximum age is Chattian (25.425 ± 2.395 Ma) from the Bovidae stem fossil.

Calibration point M: Crown Artiodactyla (70) (part of Cetartiodactyla) by Benton et al. (2015). The fossil (*Himalayacetus*) horizon, Pakistan, was correlated to a planktonic foraminifer zone that is assigned an age of 53.25 ± 0.75 Ma (Bajpai & Gingerich, 1998). Maximum age is considered at 66.0 Ma (K–Pg boundary), but I prefer 63.8 ± 2.2 Ma at node N.

Calibration point N: Crown Eulipotyphla (68) by Benton et al. (2015). Fossil *Adunator* found in strata of the lower Fort Union Formation, Bighorn Basin, USA, that is considered to be of the Danian stage (63.8 ± 2.2 Ma) as considered the stem age. Maximum age is the same as the above: the *Juramaia* age of 160.7 ± 0.4 Ma.

Calibration point O: Crown Afrotheria (63) by Benton et al. (2015). Fossil *Eritherium* was found from Morocco (Gheerbrant, 2009), from strata considered to be of the Thanetian stage (57.6 ± 1.6 Ma). The maximum age is the same as the above: the *Juramaia* age of 160.7 ± 0.4 Ma.

Calibration point P: Crown Xenarthra (62) by Benton et al. (2015). Fossil *Riostegotherium* was found from strata in Brazil (Scillato‐Yane, 1976) considered to be of the Thanetian stage (57.6 ± 1.6 Ma; Woodburne et al., 2014). The maximum age is the same as the above *Juramaia* age of 160.7 ± 0.4 Ma.

Calibration point Q: Crown Marsupialia (58) by Benton et al. (2015). Fossil *Djarthia* was found fromstrata of Murgon, Australia, considered to the Yepresian stage (51.96 ± 4.6 Ma; Beck et al., 2008). The maximum age is from fossil *Sinodelphys* (Metatheria) described from the Jehol Biota, Liaoning, northern China (Luo et al., 2003). Intercalated silicic tuff from these strata was dated by the Ar–Ar method at 130.7 ± 1.4 Ma (He et al., 2006). Note that Bi et al. (2018) reinterpreted *Sinodelphys* as an early member of Eutheria.

Calibration point R: Crown Theria (59) by Benton et al. (2015). *Juramaia sinensis* from strata from Liaoning, Northeastern China, that yielded an Ar–Ar age of 160.7 ± 0.4 Ma corresponding to the Oxfordian stage (Luo et al., 2011). Benton et al. (2015) sets the maximum age at 162.7 ± 1.1 Ma from the *Ambondro* fossil below at node S.

Calibration point S: Crown Mammalia (57) by Benton et al. (2015). *Ambondro mahabo* from Madagascar is placed within Monotremata, and the fossil‐bearing strata are considered to be the Bathonian stage (162.7 ± 1.1 Ma; Flynn et al., 1999). Maximum age is considered to be base of the Jurassic at 201.3 ± 0.2 Ma.

### 2.7 Geological event calibration

It appears that there is no conventional analysis found for MCMCTree that applies a geological event calibration. Therefore, the application example provided in this study may be specific to MEGA 11 and BEAST v1.10.4, but including BEAST v2.7. When applying a geologic calibration event, it is crucial to have substantial geological evidence and robust geochronology supporting it (c.f., Papadopoulo et al., 2010). In the case of Xenarthra (South America) and Afrotheria (Africa), their diversification is believed to have occurred due to the separation of continents during the Pangean breakup through sea floor spreading (De Baets et al., 2016). Nishihara et al. (2009) addressed this simultaneous divergence through genomic analyses, considering a geological constraint related to the breakup age of 120 Ma (De Baets et al., 2016). It is worth noting that the start date of the continental breakup at 120 Ma could be used as a calibration date. However, a potential issue arises from the possibility of animals being able to cross the nascent, very narrow proto Atlantic Ocean during that time. Therefore, the calibration age used for this geologic event should correspond to a significant physical separation of the continents and may be considerably younger than the initial rifting age of 120 Ma, considering geologic evidence of a slow initial rifting rate. For instance, it has been proposed that New World monkeys in South America rafted from Africa on floating material at a time considerably younger than 120 Ma (Houle, 1999). This highlights the importance of considering additional geological and paleontological evidence when determining appropriate calibration ages for specific geologic events.

The above issue concerning initial rifting age versus physical separation age (i.e., physical separation sufficient to isolate animals and plants) does not apply to the analysis of formation of the Ryukyu Islands (Osozawa et al., 2012). The rifting and sea floor spreading initiated and proceeded rapidly and simultaneously over a 1000 km distance, parallel to the continental margin. The geologic event age of 1.55 ± 0.15 Ma is based partly on the oldest age of marine strata that surrounding each island, recording full separation from the East Asian continental mainland and other islands, rather than initial age of rifting. As a result, vicariant speciation simultaneously started at this time and acted on each isolated island population (c.f., Kodandaramaiah, 2011). The age of MRCA at 1.55 ± 0.15 Ma serves as a robust date for geological event calibration, signifying the isolation of each island within the Ryukyu Islands (c.f., Osozawa, Kanai, et al., 2021; Osozawa, Ito, et al., 2021, Izu‐Bonin Islands). Based on Osozawa et al. (2012), I have already applied this calibration date as tMRCA of insect clades (Osozawa et al., 2013; Osozawa, Fukuda, et al., 2016; Osozawa, Kanai, et al., 2021; Osozawa, Oba, et al., 2015; Osozawa, Ogino, et al., 2016; Osozawa, Sato, et al., 2017; Osozawa, Shiyake, et al., 2017; Osozawa, Takáhashi, et al., 2015, 2017; Osozawa & Wakabayashi, 2015, 2022). These species started vicariance at 1.55 Ma and each node specific clade was calibrated at 1.55 ± 0.15 Ma. The preset study applied this geologic calibration to Asian continental and Japan island otters (Waku et al., 2016; Figures 1, 2, 3, calibration point Z; Table 1).

### 2.8 Recovering ancient base substitution rates

The resulting mammalian Bayesian Inference tree, calculated using the BEAST platform software, was visualized using FigTree. Utilizing BEAUti, I selected the relaxed clock model (Ho & Duchene, 2014), and assessed the base substitution rate over time. In the FigTree interface, the median rate of three branches, which is not constant in the case of a relaxed clock model (Drummond et al., 2006), and the node age are displayed on each node using the “Node Labels” tab. It is important to note that while FigTree can output every dated tree created by BEAST v2.7, MrBayes 3.2.7, and MCMCTree in PAML (4.9e), the rate data is not displayed using the “Node Labels” tab (only “Node ages” are displayed).

The rate‐related function in BEAST v1.10.4, employed in this study, has not been utilized in any prior publications, except for our own, inclusive of Osozawa and Wakabayashi (2022). In this study, akin to the relaxed clock model proposed by Drummond et al. (2012), I observed variable base substitution rates over time. Consequently, I created a base substitution rate diagram (“rate median” indicated at each node in FigTree) versus age (“Node age” indicated at each node in FigTree) along with its corresponding equation (insets in Figures 1 and 2) using an Excel function.

**FIGURE 4:**
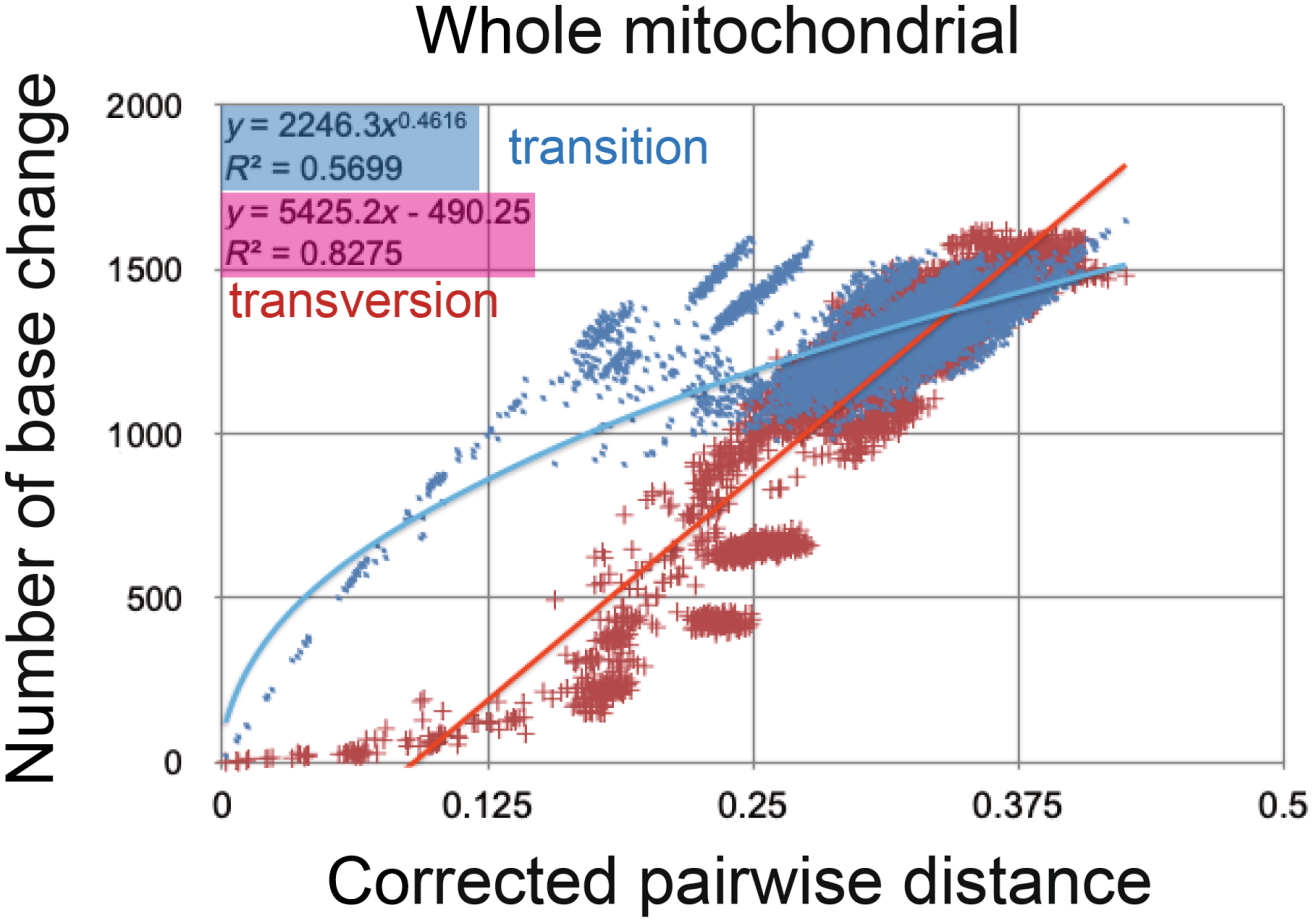
Number of base changes of transition and transversion versus corrected pairwise distance diagram for whole mitochondrial gene. When examining transitions, an exponential approximate curve can be applied, displaying a mild saturation compared to the non‐saturated transversion plots.

The inset in Figures 2 and 3 shows that the base substitution rate was relatively slow during the Cretaceous and Paleogene. This raised the question of whether the apparently slow rate reflected mutation saturation, so I examined the relation between pairwise distance and number of transitions or transversions for whole mitochondrial gene, using the additional function in MEGA 11 (Tamura et al., 2021; Figure 4). Similar approaches by alternative software are found in recent publications of Yuan et al. (2022) and Bi et al. (2023).

## 3 RESULTS

### 3.1 Use of maximum ages does not impact dating

Figures 2 and 3 were constructed by calibration by input tMRCA = minimum age, and Figures S1 and S2 were constructed by calibration by input tMRCA = (maximum age + minimum age)/2. In Figures S1 and S2, the output dates are not affected by the maximum ages and they equate to the input minimum ages as in Figures 2 and 3. These two kinds of trees are similar, unaffected by application of the two types of calibrations. A difference is longer 95% HPD (height posterior density) in Figures S1 and S2 compared to Figures 2 and 3 (these bars were not shown in these figures to avoid complexity). An excessively large and unreasonable standard deviation: (maximum age – minimum age)/2 such characterized calibration points C, F, and N ~ R, that is, overestimated maximum ages, but this had minimal impact on the timetree that was strongly controlled by the remaining points with very small standard deviations. Although a minor difference between the two methods is that Xenarthra is a sister or not a sister to Afrotheria, super‐order level differentiation occurred almost simultaneously before the K–Pg boundary for both. Klopfstein (2021) recommended the well‐dated and firmly placed fossils providing a minimum age for a particular node, and I have showed this to be a useful recommendation.

Figure S3 is a timetree made by the same BEAUti setting as Figures S1 and S2, but changed to prior lognormal distribution, whereas the dating and topology is similar to Figures S1 and S2.

### 3.2 Mammalian minimum age tree and differentiation

In original maximum parsimony tree (newick formatted tree file) made by MEGA 11, Scandentia and Eulipotyphla were not included in the major clades of Euarchontoglires and Laurasiatheria, and this topology was succeeded to the RelTime dated tree (Figure 1). MEGA 11 cannot reset these major clades, and cannot apply to the crown nodes of F 63.8 ± 2.2 Ma for Euarchontoglires and N 63.8 ± 2.2 Ma for Laurasiatheria. Consequently, super‐order level differentiation was expressed to start simultaneously at 53 Ma after the K–Pg boundary (66.0 Ma).

In BEAST v1.10.4 analyses, I can set ingroup taxa or clade in BEAUti, and Scandentia and Eulipotyphla were included in the major clades of Euarchontoglires and Laurasiatheria. I entered tMRCAs of super‐order to grand‐order level clades, except for the Glires clades, and the relationships of these super‐order clades were reasonably restored in Figures 2 and 3. Prototheria is the basal clade at 160.97 Ma, and Metatheria is next at 94.66 Ma (Figure 2). Placentalia differentiated into super‐order clades after 73.42 Ma (Figure 2), and these super‐order clades were thereafter differentiated into grand‐order–species‐level clades. The relation of grand‐order–species‐level clades was reasonably restored in Figures 2 and 3.

Placentalian super‐order level differentiation dates were estimated as occurring before the K–Pg boundary (66.0 Ma) except for Euarchontoglires clade at 65.64 Ma (Figure 2). Because prior tMRCAs of Placentalia super‐order clades were set younger than 66.0 Ma (K–Pg boundary), all the order level differentiations were post K–Pg boundary followed by the family level differentiations (Figures 2 and 3).

For the great apes (Figure 2), *Pongo* differentiated from other great apes at 13.4 Ma. *Gorilla* was differentiated from *Pan* + *Homo* at 8.0 Ma. I estimate *Homo sapiens* to have differentiated from *Pan* at 5.69 Ma (target age at node X), *Homo sapiens* ssp. *Denisova* differentiated from *Homo sapiens neanderthalensis* + *Homo sapiens* at 2.09 Ma, and *Homo sapiens neanderthalensis* was differentiated from *Homo sapiens* at 0.95 Ma.

### 3.3 Variable mammalian base substitution rate

The base substitution rate versus age diagram (Figures 2 and 3 insets) shows that the rate was not constant and has exponentially increased from ca. 20 Ma to the recent time, and the heavy red trendline and associated equation are shown. This phenomenon of variable base substitution rate is not an artifact, and there is no saturation of whole mitochondrial gene from the Cretaceous onward (Figure 4; cf., fig. 4 in Osozawa & Wakabayashi, 2022). The transitions may be apparently saturated, but if so, all the previous dated trees including mine is mislabeled for ancient times.

## 4 DISCUSSION

### 4.1 Placental ordinal radiation after the K–Pg boundary

Three diversification models of extant placental mammals were proposed with respect to the K–Pg extinction event (Archibald & Deutschman, 2001), and in short, the inter ordinal (not superordinal) diversifications were in the Paleogene (after the K–Pg boundary; explosive model), across (i.e., before and after) the K–Pg boundary (long fuse model), and in the Cretaceous (before the K–Pg boundary; short fuse model) (the clearest definition; O'Leary et al., 2013). These models were reviewed and modified by Phillips (2016), Foley et al. (2016), Liu et al. (2017), Springer et al. (2019), Upham et al. (2021), Murphy et al. (2021), and Carretero et al. (2022), as listed in Table 2, but the definitions are controversial, and causative factor of the short fuse model was not addressed.

Although super‐order level diversifications for Xenarthra, Afrotheria, Laurasiatheria, and Euarchontoglires took place prior to the K–Pg boundary at 73.42, 71.0, and 68.06 Ma, and post K–Pg 65.64 Ma, all the order level (grand‐order–infra‐order level) diversifications occurred after the K–Pg boundary (Figures 2 and 3); this supports the explosive model. Also RelTime parsimony tree (Figure 1) clearly supports the explosive model. The explosive model predicted that the ordinal radiation of present‐day mammals occurred just after the K–Pg boundary was triggered by the mass extinction event eliminating non‐avian dinosaurs and most of the end‐Cretaceous fauna.

O'Leary et al. (2013) proposed the explosive model by building a timetree from the combined phenomic and molecular parsimony analyses for fossil and living species, similar to the total‐evidence dating. They applied multiple fossil ages for the oldest members of the clades to the timetree to determine minimum divergence dates, and the fossil minimum ages directly affected their timetree. I showed that maximum ages did not affect on my timetree whereas minimum ages did (Figures 2 and 3 vs. Figures S1 and S2).

In several studies, calibration dates were after Benton et al. (2015) and the software employed was MCMCTree (Yang, 2007). The mammalian timetrees by dos Reis et al. (2012), Foley et al. (2016), Liu et al. (2017), Wu et al. (2017), Upham et al. (2019, 2021, MrBayes; considered maximum age), Murphy et al. (2021), Carretero et al. (2022), and Foley et al. (2023) accord with the long fuse model as listed in Table 2, and ordinal differentiations across the K–Pg boundary (66.0 Ma). Because the issue of ordinal differentiations taking place before or after the K–Pg boundary is critical, I checked the affect of the calibration described in dos Reis et al. (2012). Affected by marginal prior density of divergence times in MCMCtree analyses (see their Figure 3), the total of 12 node calibrations solely by minimum ages (lacking maximum ages) estimated posterior ages (posterior density in their figure 3) older than the minimum ages and closer to the presumed maximum age (predicted in MCMCTree but differing from my analysis). Therefore, these 12 node calibrations, lacking the maximum age data produced the unexpected older node age estimates that defined the long fuse model. Note that other 11 node calibrations by both minimum and maximum ages and 2 node calibrations by solely maximum age put the posterior ages between the minimum and maximum age, as I predicted (in BEAST v1. X analyses, close to the minimum ages as shown by Figures S1 and S2). MCMCTree analyses apparently require inclusion of maximum age data to avoid this disadvantage and obtain an accurate timetree, but I concur with the recommendation of Klopfstein (2021) that inflated maximum ages should be excluded from dating analyses. Application of whole or partial genome‐scale sequence data as done by dos Reis et al. (2012), Foley et al. (2016), Upham et al. (2019, 2021), Murphy et al. (2021), Carretero et al. (2022), and Foley et al. (2023), as listed in Table 2, is unrelated to the precision of node dating, for which the calibration method is basically important (Osozawa, Kanai, et al., 2021; Osozawa & Wakabayashi, 2022).

Hassanin et al. (2021) applied a lognormal distribution on the calibrated node ages and compared the result to that obtained applying a uniform distribution for the minimum and maximum age sets. In BEAST v.1 X, using minimum and maximum ages, I selected lognormal for prior distribution to construct Figure S3 and compared the result to Figures S1 and S2 constructed with prior normal distribution. My trial showed that posterior node ages that are close to the minimum ages (and unaffected by large standard deviation) were not affected by the prior lognormal distribution (Figure S3) so the distributional setting was the not the source of the difference in long fuse model node ages compared to my own.

The timetrees by Bininda‐Emond et al. (2007), Meredith et al. (2011), and Pozzi et al. (2014) support the short fuse model. The source of discrepancy compared to the my result and others may also be related to also considering the maximum ages in their dating analyses, but dos Reis et al. (2012) did not address the reason of discordance with Bininda‐Emond et al. (2007).

### 4.2 Relation to the middle Cretaceous Pangea breakup

In Eutheria (Placentalia), divergence into Afrotheria, Xenarthra, and Boreoeutheria (Laurasiatheria + Euarchontoglires) was considered to be related to large‐scale vicariant speciation triggered by the breakup of the supercontinent Pangea into the Laurasia and Gondwana (Africa and South America). The phylogenetic basal split was considered to have followed the progression of continental separation between 100 and 120 Ma (Murphy et al., 2001; c.f., De Baets et al., 2016). Nishihara et al. (2009) geologically estimated the date of separation and proposed the simultaneous divergence beginning at ca.120 Ma. The age of the fossiliferous Santana Formation, Brazil, South America, is coeval with the initial breakup of the supercontinent Gondwana and initiation of the Atlantic Ocean, and may be Aptian, Albian or possibly Cenomanian of the Cretaceous, a rather loosely constrained date of ca.125–95 Ma (Martill, 2007). Foley et al. (2023) accepted the vicariance from their whole genome analyses, although the dates were overestimated when using MCMCTree. Additionally, the root age was calibrated at 102 Ma, based on the estimated Pangea splitting date using node dating, which is not derived from fossil evidence and can be considered as an example of typical circular reasoning.

Reports of dos Reis et al. (2012) and O'Leary et al. (2013) rejected the possibility of vicariance; their crown age for Eutheria (Placentalia) was less than 90 Ma, younger than above 125–95 Ma breakup. My data also appears to preclude a connection of the vicariance to the Pangea breakup; my crown ages for Eutheria (Placentalia; Figure 2) are 73.42 Ma, 57.26 Ma for Xenarthra, and 57.44 Ma for Afrotheria, much younger than the breakup age. However, trigger for Placentalia superordinal differentiations beginning at 73.42 Ma (Figure 2) has been remained unsolved.

The initial rifting and seafloor spreading was complex process that may explain why the differentiation age postdates the initiation of rifting but may still be related to sea floor spreading and physical separation of the continents (c.f., De Baets et al., 2016). Marine magnetic anomalies on the Atlantic Ocean floor clearly show the sporadic and somewhat slow nature of the early history of the opening of this ocean. The configuration at Chron34 (84 Ma) after the Cretaceous magnetic quiet zone (long normal polarity epoch; superchron K‐T at 118–84 Ma) was shown by Moulin et al. (2010), and the south Atlantic Ocean had spread to a width of over 500 km (minimum distance between Africa and South America) at Chron 34 (84 Ma). Because the ocean or seaway was much narrower before then, 84 Ma may be considered to be a starting date of continent level vicariance and divergence between Afrotheria and Xenarthr, which might have triggered the Placentalia superordinal differentiations shown in Figures 2 and 3.

The splitting date of Lorisiformes (Africa and Southeast Asia) and Lemuriformes (Madagascar) was estimated at 36.33 Ma (Figure 2), and unrelated to the initial breakup, but Lemuriformes in Madagascar are considered to have arrived by oceanic dispersal from Africa (Horvath et al., 2008). I estimated divergence time of New World monkeys and Old World monkeys at 35.58 Ma (Figure 2), and the probable dispersal mechanism was proposed to be rafting on floating debris from Africa to South America (Houle, 1999).

The Panama arc had docked and merged with the Andean arc during collision with South America (Farris et al., 2017), and the Central American Seaway that accommodated Caribbean‐Pacific water exchange closed as a result of being blocked by the formation of the Panama isthmus (De Baets et al., 2016). The date of closure of this seaway was indirectly assumed to be the late Oligocene (25 Ma) from the exhumation ages of rocks indicated by (U‐Th)/He and fission‐track thermochronology coupled with change of geochemistry of Panama arc magmatism (Farris et al., 2011). Alternatively, the date of seaway closure was estimated to be middle Miocene (13–15 Ma) from U–Pb ages of magmatic detrital zircons included in the northern Andean frontal basin (Montes et al., 2015), and to be the middle Miocene (18 Ma) from Ar–Ar ages of ignimbrite (Buchs et al., 2019). This date is relevant to the Great American Biotic Interchange and the vicariance of shallow marine organism between the Atlantic and Pacific Oceans (Bacon et al., 2015). They demonstrated significant waves of dispersal of terrestrial organisms at approximately ca. 20 and 6 Ma and corresponding events separating marine organisms in the Atlantic and Pacific oceans at ca. 23 and 7 Ma.

Divergence of Laurasiatheria (assumed to have originated in North America; Laurentia) in Figures 2 and 3 may be related to the partial Pangea breakup into North and South America, that is, initial formation of the Central American Seaway. Marine magnetic anomalies on the Atlantic Ocean floor constrain ancient relative position of North and South America, but the present Caribbean lithosphere obscures the history of the Central America Seaway. According to Pindell et al. (2011), the proto Greater Antillean magmatic arc was formed along the Pacific side of Central America by entrapping the Caribbean seaway and connecting North and South America at ca. 130 Ma, and the seaway was always narrow (maximum: 1000 km), constrained by the present position and distance of 1000 km between North and South America. The Panama arc is younger than 60 Ma (Montes et al., 2015), and the arc nearly connected to South America. Therefore the Central American Seaway was not wide enough to separate Laurasiatheria and trigger vicariance (c.f., Figures 2 and 3).

Regarding evolution of Euarchontoglires (assumed to have originated in Europe‐Eurasia) relative to Laurasiatheria (assumed to have originated in North America), the northern Atlantic Ocean aided by the Iceland hotspot began the latest episode of spreading at chron 24 (53.3 Ma) after multiple earlier phases of extension (Barnett‐Moore et al., 2018). The seaway was narrow before chron 24, so vicariance was not triggered in the proximal continental masses (c.f., Figures 2 and 3).

When considering the positioning of Euarchontoglires in relation to Afrotheria, Atlantic marine magnetic anomalies provide constraints on the paleo‐position of Europe and Africa. The Gibraltar strait is a significant feature, serving as the pivot point of the western margin of these continents. The Mediterranean Sea and the older Neotethyan Ocean terminated or narrowed at their western apex at the Gibraltar strait, which narrowly separated Europe and Africa, although not continuously, as this narrow seaway experienced periodic closures. Consequently, the origin of Euarchontoglires cannot be solely explained by vicariance (Figures 2 and 3). It is important to note that the closure of the Neotethyan Ocean is intricately linked to the formative processes of both the European Alps and the Himalayan Range. The former was shaped by the continental collision between Europe and Africa, as evidenced by late‐stage exhumation around 15 Ma, indicated by apatite and zircon fission track ages (Bertrand et al., 2017). Similarly, the latter was influenced by the collision between the Indian‐Arabian and Eurasian Plates, with exhumation facilitated by the South Tibetan Detachment System at approximately 20 Ma (Searl & Godin, 2003). Other geological features in different regions also mark this significant event, such as gneissose and granitic domes in northern Vietnam around 25 Ma (Osozawa, Vuong, et al., 2015) and Zagros suture granitoids with a U–Pb age of 37.7 ± 1.0 Ma (Rezaei et al., 2021). The timing of the Neotethys closure might be linked to the dispersal of Afrotheria or Euarchontoglires. Regarding Euarchonta, a component of Euarchontoglires, Scandentia (Southeast Asia) and Dermoptera (Southeast Asia) are the oldest lineages (c.f., Figures 2 and 3), Lorisiformes (Africa and Southeast Asia) is a sister to Lemuriformes (Madagascar) as noted, and Tarsiiformes (Southeast Asia) is a sister to Simiiformes including New World monkeys (Figures 2 and 3). For my calibration using the crown Primates date (81) by Benton et al. (2015), the oldest fossil Primate was from Thanetian strata of Morocco, dated at 57.6 Ma. Euarchonta originated in Southeast Asia and Africa, but the origin of Euarchontoglires is not clear.

In Metatheria, *Dromiciops gliroides* is known from Patagonia, South America, but included in Australidelphia. In the timetree by dos Reis et al. (2012), this species occupied the basal lineage of Australidelphia (pre K–Pg), and a sister relationship to *Notoryctes typhlops* (21.27 Ma in Figure 2; 33.12 Ma in Figure S1). Nilsson et al. (2010) showed a single marsupial migration from South America to Australia by their phylogenetic study including *D. gliroides*. Because the Scotia plate spreading initiated after chron C11n (30 Ma; Riley et al., 2019) to form the Drake Passage, Patagonia, the Scotia arc, and the Antarctic Peninsula were connected to form long land bridge. Therefore, the marsupial migration was via Antarctica through the long bridge before 33 Ma, and dispersed in Australia before 33 Ma when the Tasman Gateway began opening (Lagabrielle et al., 2009).

I used a tMRCA of Asian continental and Japan island otters of 1.55 Ma, an age that reflects the isolation of the Japanese islands from the Korean peninsula by the Tsushima strait (Osozawa et al., 2012) which triggered vicariant speciation on isolated populations in Japan. This process is unrelated to dispersal to Japan by a land bridge and subsequent vicariance. Otters found on the Tsushima Island in 2017 probably dispersed there from the Korean peninsula by swimming. Similarly, I did not use a calibration age of 1.55 Ma for the wild cat (*Prionailurus bengalensis euptilurus*) on the Tsushima Island (lacking on the Japan main islands), because relatively recent dispersal from Korea is plausible.

### 4.3 Relation to the middle Cretaceous angiosperm radiation

Angiosperms diversified and radiated in middle Cretaceous time (Magallon et al., 2015; Osozawa, Nakejima, et al., 2021; around 125 Ma), and beetles have been shown to have contemporaneously co‐radiated around middle Cretaceous time (McKenna et al., 2015). Mammalian ordinal radiation took place after the K‐P boundary (66.0 Ma), and was unrelated to the middle Cretaceous Angiosperm radiations (cf., Benton et al., 2022). I do not refer to possible co‐radiation crossing the K‐P boundary by Benton et al. (2022).

### 4.4 Expanded C4 grasslands and Catarrhini radiation

Calibration of crown Catarrhini was from the African fossil age of 24.93 ± 0.49 Ma (Figures 1 and 2 node D; Roberts, O'Connor, et al., 2010; Roberts, Stevens, et al., 2010). Stevens et al. (2013) suggested a possible link between diversification of Catarrhini and development of the prominent East African rift system, uplift of the African plateau, and the consequent climate and environmental changes. If this is correct, Catarrhini was generated in East Africa. However, Hylobatidae and fossils are known from Southeast Asia, including China (Benton et al., 2015).

Uplift of East Africa led to a drastic reorganization of atmospheric circulation, and this caused strong aridification since ca. 8 Ma (Sepulchre et al., 2006). The consequent progressive increase of open grassland may have been linked to African Hominidae evolution. I estimated crown age of gorilla at 8.0 Ma as calibrated by point B of 8.072 ± 0.8 Ma (Figure 2). This is consistent with the hypothesis of in situ African evolution of the *Gorilla*–*Pan*–*Homo* clade (Katoh et al., 2016). It is worth noting, however, that Ponginae is native to the rainforests of Indonesia and Malaysia and a *Sivapithecus* (Ponginae) fossil was found from Pakistan (Benton et al., 2015).

The Quaternary glaciations may have been triggered by expansion of land grasses (Poales), because this process increased carbon fixation that consequently decreased atmospheric CO_2_ concentration (Taira, 2007). C4 plants are efficient in CO_2_ fixation (Taira, 2007), and C4 Poales plants appeared and began diversification at 17.95 Ma (C4 dicots at 23.15 Ma) based on our Angiospermae timetree (Osozawa, Nakejima, et al., 2021). Carbon isotope ratios from mammalian fossil tooth enamel show that dietary change to include C4 plants started at 9.9 Ma in eastern Africa (Uno et al., 2011). In addition, mammalian fossil teeth from the sub‐Himalayan Siwalik Group, Pakistan, show that C4 savanna replaced C3 forest and woodland between 8.5 and 6.0 Ma (Badgley et al., 2008). The expansion of C4 grasses was a global phenomenon that also included North America and South America, beginning in the late Miocene and persisting to the present day, including the present glacial–interglacial period (Cerling et al., 1997). Rodentia in Argentina may have been triggered by the expansion of relatively open and arid environments that arose near the Miocene‐Pliocene boundary (Candela et al., 2019). This event may have impacted Hominidae radiation as well as Laurasiatheria, such as Bovidae and Equidae in tropical savannah and temperate prairies and steppe (Taira, 2007).

I estimated the date of the human–chimpanzee speciation event at 5.69 Ma (Figure 2), which is close to the estimate of ca. 6 Ma by Scally et al. (2012) but younger than ca. 7 to 8 Ma by Langergraber et al. (2012). I estimated *Homo sapiens* ssp. *Denisova* speciation event at 2.09 Ma, and *Homo sapiens neanderthalensis* speciation event at 0.95 Ma (Figure 2), although these are older than 0.77 to 1.3 and 0.32 to 0.61 estimated by Krause et al. (2010), 0.65 to 0.97 Ma and 0.32 to 0.48 Ma estimated by Fu et al. (2013), and 0.72 to 1.41 Ma and 0.36 to 0.46 Ma estimated by Posth et al. (2016).

### 4.5 Increasing base substitution rate toward recent times: Implications for biodiversity expansion and the emergence of humans

Figures 2 and 3 insets show that the base substitution rate has varied through time, strongly refuting the premise of a constant molecular clock (strict clock model). The data may possibly suggest a mild peak in base substitution rates in the ca. 70–50 Ma age range that may possibly reflect mammalian radiation after the K–Pg boundary following the explosive model (c.f., Foley et al., 2023). Although Bininda‐Emond et al. (2007) showed diversification rates through the time with a peak at 93 Ma, their estimates reflected their short fuse model.

The general trend of base substitution rates (insets in Figures 2 and 3) appears to exhibit an exponential increase in rates, with the most prominent rise occurring within the last 20–30 Ma (c.f., Hedges et al., 2015; Upham et al., 2019, 2021). This phenomenon was initially observed in primates (Ho et al., 2005), where it was found that using a Quaternary date calibration resulted in a faster rate than with an older date calibration. BEAST v1.10.4 allows for calibration by simultaneously applying multiple calibration points as MEGA 11 (Figure 1). However, Ho et al. (2005) utilized BEAST v1.3 (Drummond & Rambaut, 2003), which only allowed calibration of one point per run. In my study, I calibrated instantaneously in a single run using multiple dates ranging from the Jurassic to the Quaternary. Therefore, the observed increase in rates toward recent times is not an artifact of a single Quaternary date calibration, as was the case for Ho et al. (2005). It is important to highlight that in older versions of BEAST, combined gene analyses were not feasible, necessitating the definition of the rate for each gene. Consequently, even though their trendlines and equations resemble mine, their timetrees do not depict time‐dependent base substitution rates, instead assuming a constant rate consistent with a strict molecular clock.

Scally et al. (2012) suggested that base substitution rate decreased for Hominidae in stark contrast to my analysis, but they pointed out that the evolution was accelerated for African Hominidae. A human timetree calibrated by radiometric carbon data (maximum resolvable age of ca. 60 kyr) suggested that the base substitution rate was approximately 1.6‐fold higher than the older fossil‐calibrated tree (Fu et al., 2013), similar to high estimates rates obtained in studies of pedigrees and laboratory mutation‐accumulation lines (Ho et al., 2011).

Exponentially increasing base substitution rate may be expected to increase biodiversity as shown by diversified species in Figures 1, 2, 3 (c.f., Upham et al., 2019, 2021). Species diversity including human emergence may have been a consequence of extensive adaptive radiation triggered by expansion of C4 grasses, effective decreased atmospheric CO_2_ concentration, global cooling, and severe climatic change of start of the Quaternary glacial–interglacial cycles.

## 5 CONCLUSION

The analysis of a robust minimum age tree reveals the radiation of Placentalia following the Cretaceous‐Paleogene (K–Pg) boundary, accompanied by a subsequent extensive radiation linked to an exponential increase in the base substitution (mutation) rate, including the emergence of *Homo sapiens*. This Neogene radiation could potentially be attributed to the expansion of C4 grasses, leading to a decrease in atmospheric CO_2_ levels, global cooling, and the initiation of Quaternary glacial–interglacial cycles.

## AUTHOR CONTRIBUTIONS

**Soichi Osozawa:** Conceptualization (lead); data curation (lead); formal analysis (lead); funding acquisition (lead); investigation (lead); methodology (lead); project administration (lead); resources (lead); software (lead); supervision (lead); validation (lead); visualization (lead); writing – original draft (lead); writing – review and editing (lead).

## FUNDING INFORMATION

This research was partly supported by Grants‐in‐Aid for Scientific Research Japan, “Extrusion Wedge of the Sambagawa High P‐T Metamorphic Rocks,” grant number 20540441.

## CONFLICT OF INTEREST STATEMENT

The author declares that there are no competing interests.

## Supporting information


Figure S1

Figure S2

Figure S3

Appendix S1

## Data Availability

Whole mitochondrial sequence data applied, including *Homo sapiens*, were from GenBank /DDBJ. The accession numbers can be found in Figures 1, 2, 3.
