# Supplementary figures and images for: Geologically calibrated mammalian tree and its correlation with global events, including the emergence of humans
Source: Ecol Evol. 2023 Dec 19;13(12):e10827. doi: 10.1002/ece3.10827 (PMC10728886; doi:10.1002/ece3.10827)

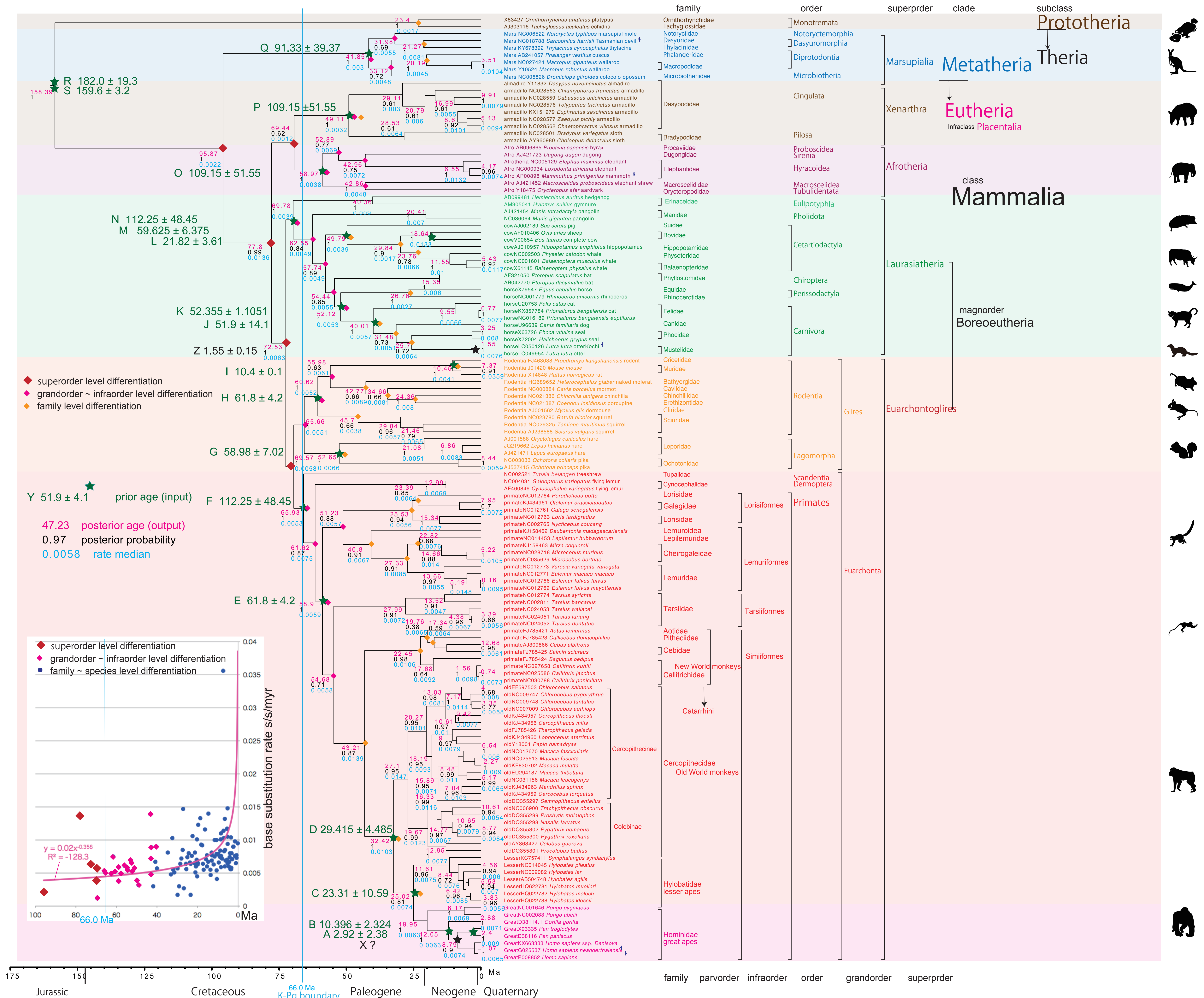

Supplement: Supplementary file 1 — Figure S1 [file ECE3-13-e10827-s004.pdf]

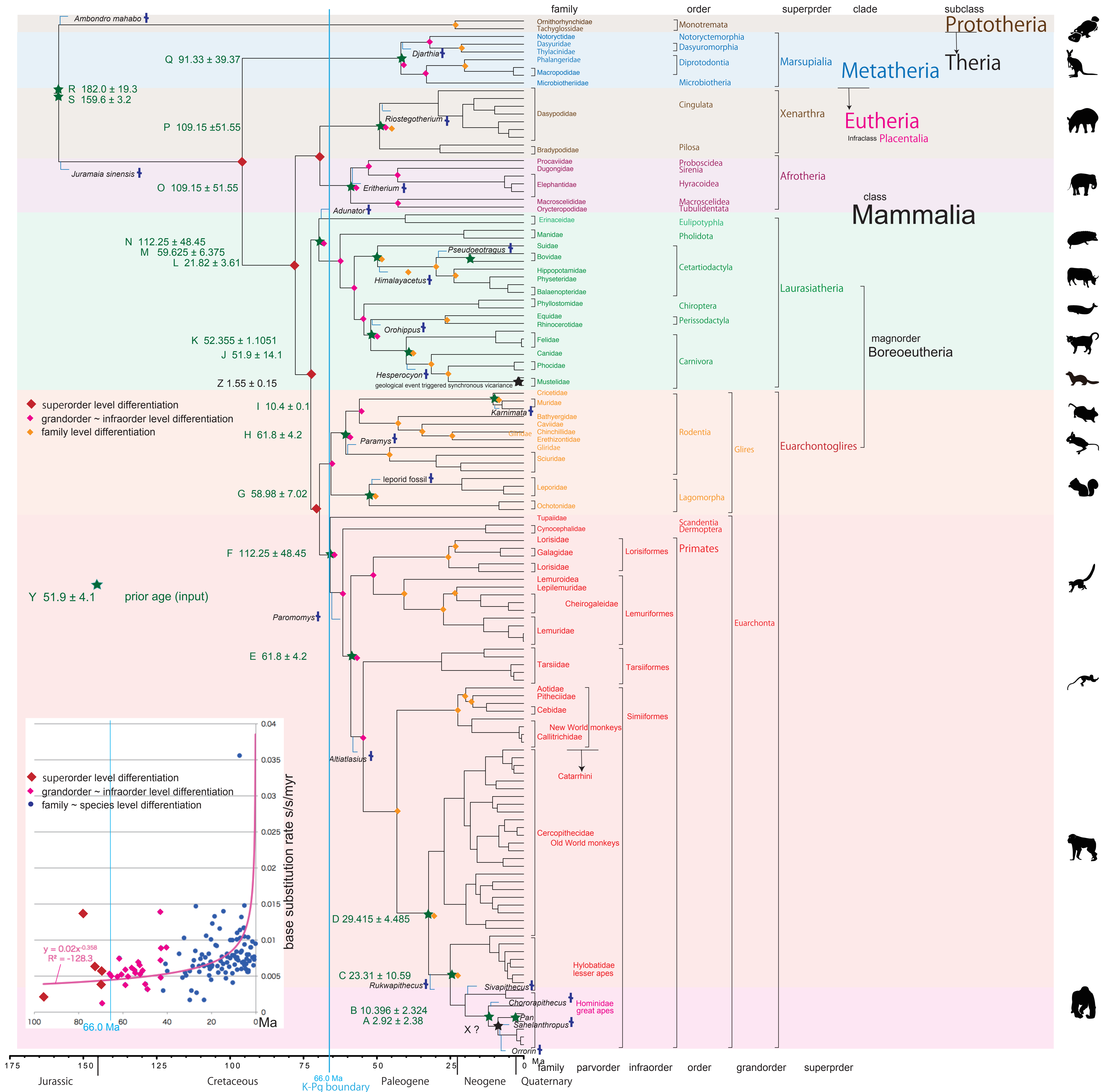

Supplement: Supplementary file 2 — Figure S2 [file ECE3-13-e10827-s001.pdf]

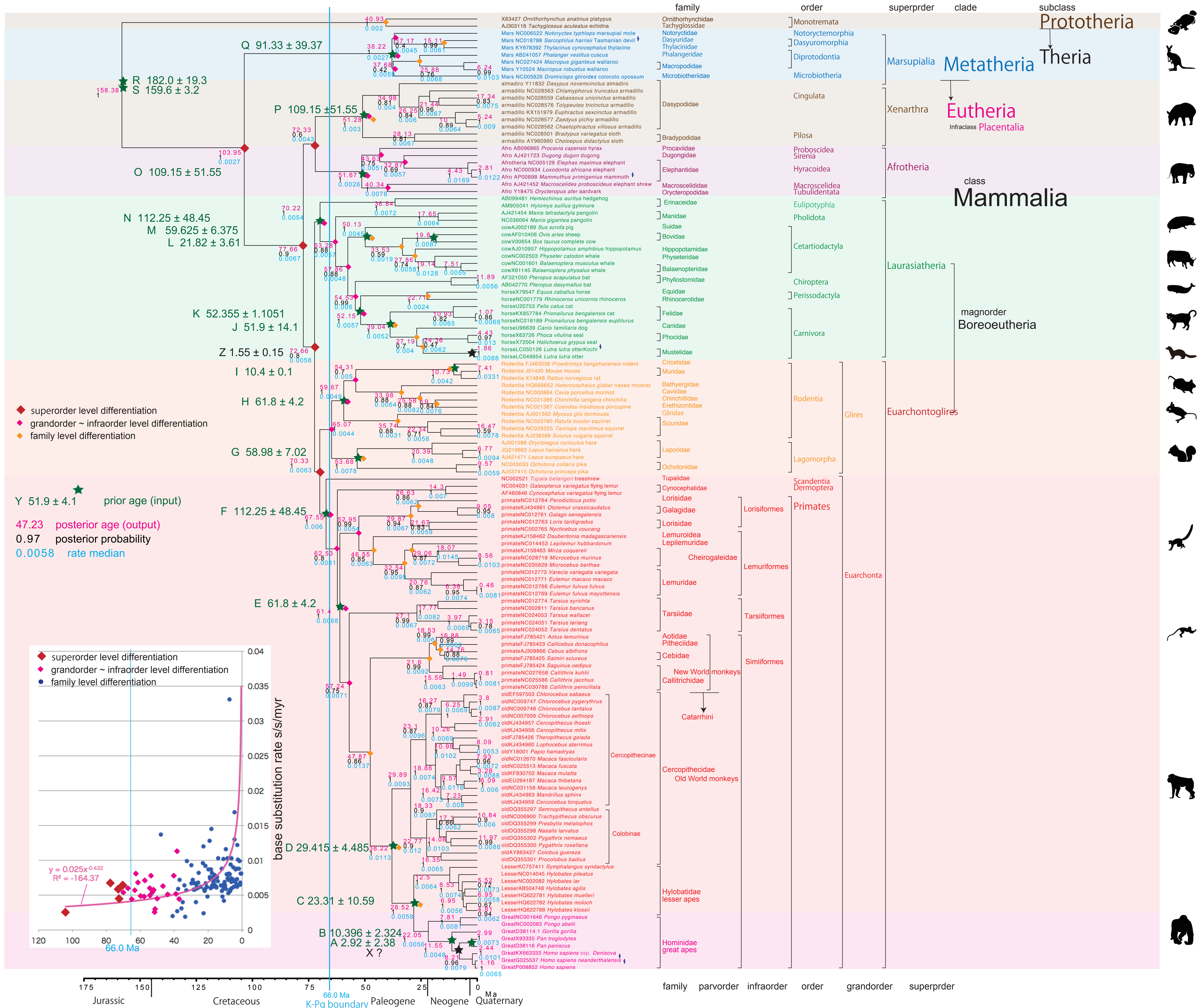

Supplement: Supplementary file 3 — Figure S3 [file ECE3-13-e10827-s003.pdf]
