# Supplementary material for: Geologically calibrated mammalian tree and its correlation with global events, including the emergence of humans
Source: Ecol Evol. 2023 Dec 19;13(12):e10827. doi: 10.1002/ece3.10827 (PMC10728886; doi:10.1002/ece3.10827)
Supplement: Supplementary file 4 — Appendix S1 [file ECE3-13-e10827-s002.docx]

**Figure S1.** Mammalian timetree built by BEAST v1.10.4. Prior distribution: normal. Fossil calibrations from A to S consider maximum ages in addition to minimum ages. *Pan*-*Homo* splitting date was not calibrated, but expressed as X, and estimated at 8.79 Ma. Geological event calibration: point Z. Ordinal differentiation was post K-Pg boundary event with an exception. Illustrations were downloaded from silhouette AC. Inset: Base substitution rate (= rate median shown at each node; mutations per bp per million years) vs age (= posterior age shown at each node) diagram. Red curve with equation: trendline drawn by Excel function.

**Figure S2.** Mammalian timetree built by BEAST v1.10.4, simplified from Fig. S4. Fossil ages with cross marks to adjust minimum ages (Benton et al., 2015) are shown close to each calibration point.

**Figure S3.** Mammalian timetree built by BEAST v1.10.4 similar to Figs. S4 and S5, but with prior distribution lognormal. *Pan*-*Homo* splitting date was not calibrated, but expressed as X, and estimated at 8.21 Ma.
